# Implications of Sample Preparation Methods on the MALDI-TOF MS Identification of Spore-Forming Bacillus Species from Food Samples: A Closer Look at *Bacillus licheniformis*, *Peribacillus simplex*, *Lysinibacillus fusiformis*, *Bacillus flexus*, and *Bacillus marisflavi*

**DOI:** 10.1021/acsomega.3c04354

**Published:** 2023-09-13

**Authors:** Daria Janiszewska, Michał Złoch, Paweł Pomastowski, Małgorzata Szultka-Młyńska

**Affiliations:** † Department of Environmental Chemistry and Bioanalytics, Gagarina 7, 87-100 Torun, Poland; ‡ Centre for Modern Interdisciplinary Technologies, Nicolaus Copernicus University, Wilenska 4, 87-100 Torun, Poland

## Abstract

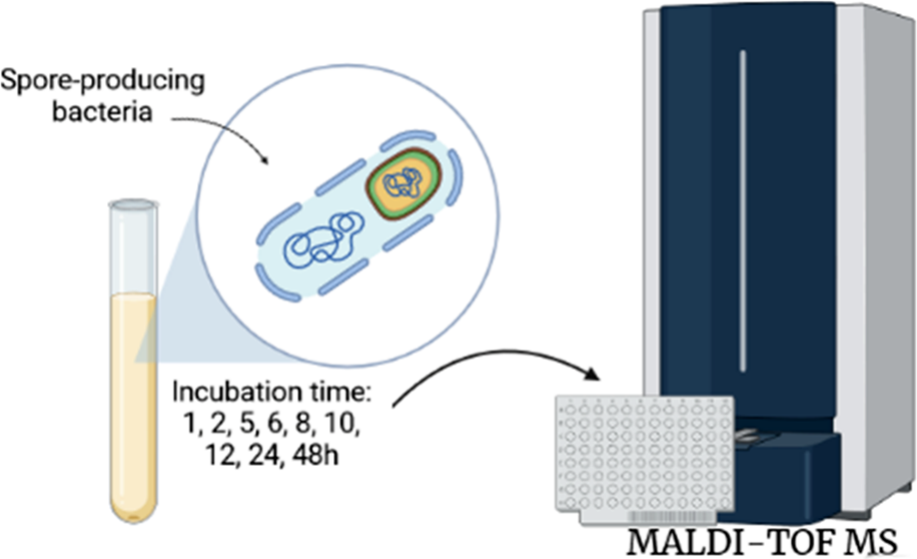

This research underscores the criticality of tailored culture conditions and incubation periods for effective and accurate identification of spore-forming bacteria: *Bacillus licheniformis*, *Peribacillus simplex*, *Lysinibacillus fusiformis*, *Bacillus flexus*, and *Bacillus marisflav*, isolated from food samples, utilizing the MALDI-TOF MS technique. All isolated strains were confirmed as Gram-positive bacteria from diverse genera through 16S rDNA gene sequencing. To enhance the accuracy of the identification process, the study employed an optimization strategy involving a varied incubation time (ranging from 1 to 48 h) and two distinct sample preparation approaches—direct transfer facilitated by formic acid and protein extraction via ethanol. It was observed that matrix-assisted laser desorption ionization–time-of-flight mass spectrometry (MALDI-TOF MS) could successfully identify approximately 47% of the samples following a 24 h incubation period. The study emphasizes the critical role of sample preparation methods in enabling precise bacterial identification. Our findings reveal the necessity of tailoring the incubation time for each sample, as the optimum period for accurate identification fluctuated between 1 and 12 h. Further demonstrating the interplay between incubation time and spore quantity, our study used the Schaeffer–Fulton staining method to show that the lowest spore counts were detected between 5 and 8 h of incubation. This provides evidence that spore formation impacts bacterial identification. Our research thus deepens the understanding of spore-forming bacteria identification using MALDI-TOF MS and illuminates the various factors affecting the dependability and accuracy of this technique. Future research may explore additional variables, such as the effect of varying culture media, to further augment identification accuracy and gain a holistic understanding of spore-forming bacterial behavior in food samples. By enhancing our knowledge, these findings can substantially contribute to improving food safety and quality assurance strategies by enabling the more accurate and efficient identification of spore-forming bacteria in the food industry, thereby elevating the standards of food safety.

## 1 Introduction

Spore formation by bacteria results from the influence of adverse external factors and drastic environmental changes. Spores are one of the survival strategies of bacterial cells. Thanks to the metabolic activity of their vegetative forms, spore-forming bacteria can be used as a biological weapon or cause opportunistic infections. Spore-forming bacteria constitute a major problem for the food industry, especially for dairy products.^1^*Bacillus* spoilage bacteria are of particular concern, as their spores are extremely resistant to most environmental stresses, including pasteurization and other heat treatments commonly used in food processing.^2,3^ Besides the dairy industry, *Bacillus* spores were isolated from various types of food, such as rice, eggs, pasta, and honey.^4−7^ The presence of *Bacillus* spores in many food industries is a consequence of several factors. For example, spores in dairy products result from the initial contamination of raw milk and subsequent temperature abuse during transport and distribution.^8^ The widespread disclosure of *Bacillus cereus* spores in cooked rice and meat may result from slow cooling and prolonged storage at room temperature, which facilitates the formation of spores.^9,10^ It was also proven that including cracked and contaminated eggs and limited disinfection can lead to the occurrence of *B. cereus.*(11) Moreover, species such as *B. cereus*, *B. thoracic*, and, to a lesser extent *Bacillus subtilis*, are pathogenic in humans and other mammals. Literature data also indicate that some strains of different species, including *Bacillus licheniformis*, *Bacillus thuringiensis*, and *Bacillus pumilus*, can cause foodborne illness (e.g., gastrointestinal symptoms that are self-limiting) typical of either *B. cereus* or *B. subtilis.*(12) The steps involved in the spore formation include DNA segregation, septum formation, engulfment, spore formation, formation of spore protein layers, cortex, membranes, and spore coat, and the maturation of the spore before mother cell lysis and release. After the endospore formation, it can remain dormant and survive in adverse environmental conditions without moisture and nutrients due to the protective structure and properties of the endospore.^13^ Moreover, endospores have different protein expressions than vegetative cells.^14^

Mesophilic spore-forming bacteria belong to two taxonomic groups: the order *Bacillales* (aerobic) and the genus *Clostridium* (anaerobic). They can both spoil organisms and pathogenic bacteria.^15,16^ The family *Bacillaceae* covers 19 genera, including *Bacillus*, *Lysinibacillus*, and *Peribacillus*.^17,18^

The MALDI-TOF MS technique is routinely used for rapid identification of microorganisms. Currently, one of the prevailing trends is to reduce the incubation time of bacteria and thus speed up analyses. Longer incubation times for *Bacillaceae* family bacteria lead to nutrient depletion in the culture medium, promoting endospores’ growth. The thick peptidoglycan walls of endospores can make identifying spore-forming microorganisms much more difficult. However, literature data indicate that this technique can locate spore-forming bacteria of the Bacillus genera.^19,20^

The research aimed to optimize the culture conditions for spore-forming bacteria isolated from food samples: sauerkraut, pickled cucumbers, and cow’s milk. In the present study, the effect of incubation time on the development of spores along the level of identification using the MALDI-TOF MS technique was investigated. In addition, two sample preparation methods were compared—the extraction of bacterial proteins by the ethanol/formic acid method and extended direct transfer involving the analysis of whole bacterial cells treated with formic acid.

## 2 Results

### 2.1 16S rDNA Bacteria Identification

Based on 16S rDNA gene sequencing, it was possible to identify all isolated strains classified as Gram-positive bacteria (Table 1). The isolates were represented by bacteria of the genera Bacillus (7NN), *Peribacillus* (8NN), *Lysinibacillus* (11NN), *Priesta* (12NN) and *Rossellomorea* (13NN). Members of *Peribacillus*, *Priesta*, and *Rossellomorea* are species until recently included in the *Bacillus* genus, which was restricted to species closely related to *B. subtilis* and *B. cereus.*(17) All of the identified bacteria are characterized by their ability to produce endospores. For the sample 7NN, sequencing showed two Bacillus species (*B. licheniformis*, *Bacillus haynesii*) with the same percent of identity (Per. Ident). The sample 8NN was identified as *Brevibacterium-frigoritolerans* and *Peribacillus simplex*. Due to slight differences in 16S rDNA sequences (≥0.5%), the species differentiation of *Lysinibacillus* was impossible.

**Table 1:** Result of Bacteria Identification Based on 16S rDNA Gene Sequencing

strain	related species from NCBI [accession number]	identity [%]	given accession number
7NN	*Bacillus licheniformis* strain DSM 13 [NR_118996.1]	99.64	OM371088
	*Bacillus licheniformis* strain BCRC 11702 [NR_116023.1]	99.64	
	*Bacillus haynesii* strain NRRL B-41327 [NR_157609.1]	99.64	
8NN	*[Brevibacterium] frigoritolerans* DSM 8801 [117474.1]	99.72	OM371091
	*Peribacillus simplex* NBRC 1570 = DSM 1321 [NR_112726.1]	99.58	
	*Peribacillus simplex* LMG 11160 [NR_114919.1]	99.58	
11NN	*Lysinibacillus pakistanensis* strain NCCP-54 [NR_113166.1]	98.46	OM372597
	*Lysinibacillus macroides* strain LMG 18474 [NR_114920.1]	98.2.5	
	*Lysinibacillus fusiformis* strain DSM 2898 [NR_042072.1]	98.25	
	*Lysinibacillus fusiformis* strain NBRC 15717 [NR_112628.1]	98.25	
12NN	*Priestia flexa* strain NBRC 15715 [NR_113800.1]	99.65	OM372596
	*Priestia flexa* strain IFO15715 [NR_024691.1]	99.65	
13NN	*Rossellomorea marisflavi* strain TF-11 [NR_119437.1]	99.86	OM372574

### 2.2 MALDI-TOF MS Bacteria Identification

Bacteria identification from food samples was performed after applying 3 sample preparation approaches: direct transfer, on-target extraction, and in-tube extraction. Using these three methods, identification was obtained after 24 h of bacterial incubation for 46.67% (7/15) of samples (Table 2). The identification at the genus level (score 1700–1999) using a direct transfer and on-target extraction was obtained for only two samples, 8NN and 13NN. For the sample 12NN, the identification was obtained only for on-target extraction. Performing protein extracts allowed the identification of microorganisms at the genus level in the samples 11NN and 12NN.

**Table 2:** Results Obtained for Microflex LRF MALDI-TOF MS Analysis^a^

		microflex LT MALDI-TOF MS					
MALDI identification results	sample name	direct transfer	score	on-target extraction	score	in-tube extract	score
pickled cucumbers	7NN						
sauerkraut	8NN	*Peribacillus simplex*	1.96	*Peribacillus simplex*	1.80		
cow milk	11NN					*Lysinibacillus fusiformis*	1.96
cow milk	12NN			*Bacillus flexus*^1^	2.13	*Bacillus flexus*^1^	1.86
sauerkraut	13NN	*Bacillus marisflavi*^2^	1.94	*Bacillus marisflavi*^2^	2.04		

a The probability of correct identification in the MALDI Biotyper 3.0 system was expressed in the form of a point index and a graphical index: 2.300–3.000: reliable identification of the microorganism up to species level; 2.000–2.299: reliable identification of the microorganism to the genus level and the probable result of identification to species level; 1.700–1.999: probable result of identification to the level of the genus; ≤1.699: not reliable identification result. New nomenclature: ^1^*Priesta flexa*, ^2^*Rossellomorea marisflavi*.

On-target and in-tube extraction were selected for further analysis as sample preparation methods for the MALDI-TOF MS analysis. No reliable identification (score <1700) was obtained for the remaining samples, as shown in gray in the table.

### 2.3 Selection of the Incubation Time

Regarding the selection of the incubation time of spore-producing bacteria, the results are summarized in Table 3. Additionally, the dependence of bacterial identification score values on incubation time was plotted.

**Table 3:**
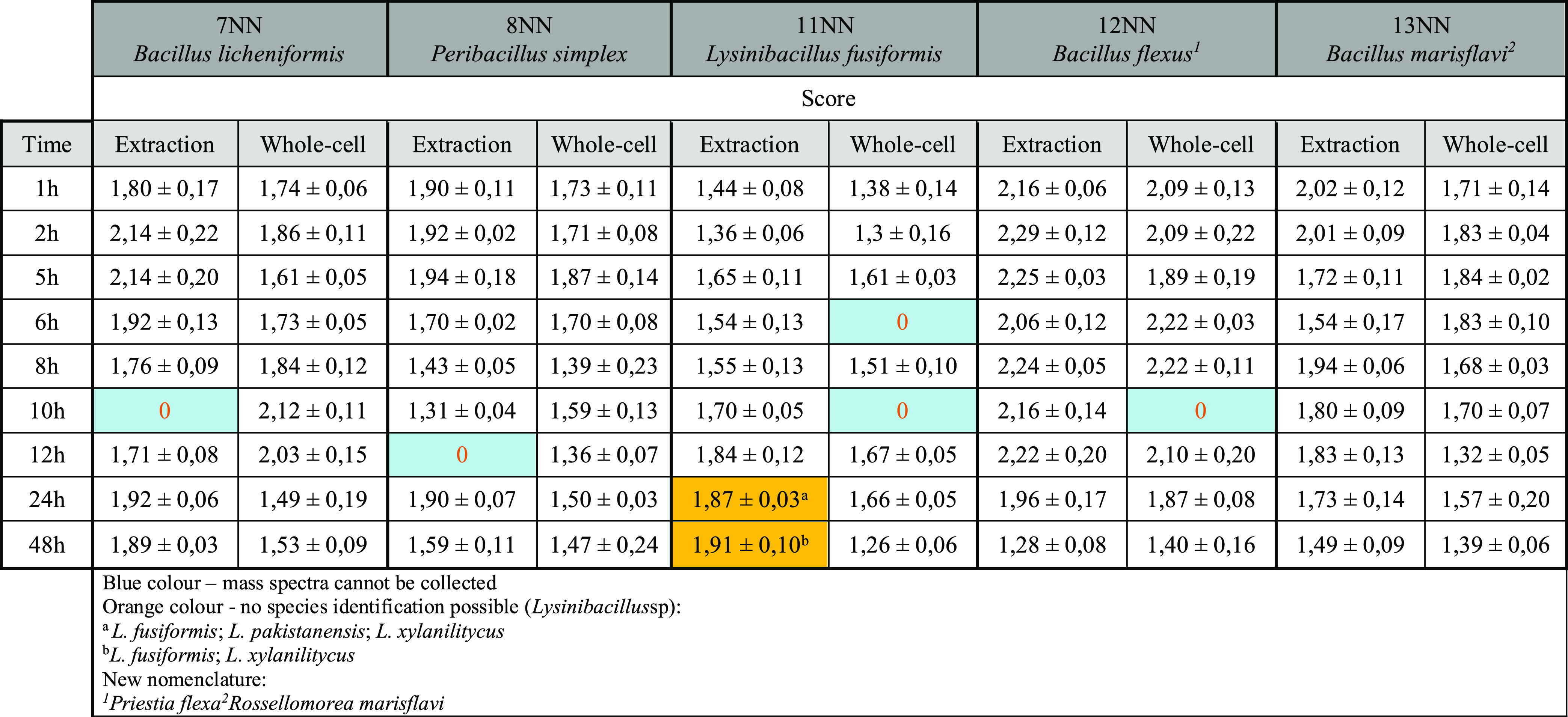
Results of Bacterial Identification Using MALDI-TOF MS Including Incubation Time and Sample Preparation Method

a Blue color—mass spectra cannot be collected. Orange color—no species identification possible (*Lysinibacillus sp*): ^a^*L. fusiformis*; *L. pakistanensis*; *L. xylanilitycus*. ^b^*L. fusiformis*; *L. xylanilitycus*. New nomenclature: ^1^*Priestia flexa*^2^*Rossellomorea marisflavi*.

The best results were achieved for the sample 12NN, where *Priesta flexa* (*B. flexus*) was identified at the species level as early as 1 h of incubation, and satisfactory identification results were achieved up to 24 h of incubation. One-hour incubation also allowed the identification at the bacterial genus level in the samples 7NN, 8NN, and 13NN, where additionally, the sample preparation by extraction allowed the identification at the species level. Isolate 7NN was identified in 73.68% (14/19) of the cases, of which 35.71% (5/14) identifications were at the species level. *P. simplex* (8NN) was identified between 1–6 h of incubation, with the extract allowing the identification at 5 h, where the score ≥2. In this case, 52.63% (10/19) of the analysis gave reliable identification results, of which 10% (1/10) were conducted at the species level.

The identification of sample 11NN at the genus level was possible after 5 h of incubation and protein extract preparation. Between 10 and 24 h, bacteria were identified at the genus level. However, species identification was impossible because successive replicates resulted in different species of bacteria belonging to the genus *Lysinibacillus:L. xylanilyticus*, *L. fusiformis*, and *L. pakistanensis*. At the species level, *Lysinibacillus* was identified after 48 h of incubation, but again two species were obtained from several replicates: *L. xylanilyticus* and *L. fusiformis* (orange color in Table 3). *Rossellomorea marisflavi* (*B. marisflavi*) in the sample 13NN was identified after 1–6 h of incubation. However, the best results were obtained for the extraction method after 1 and 8 h of incubation.

**Figure 1:**
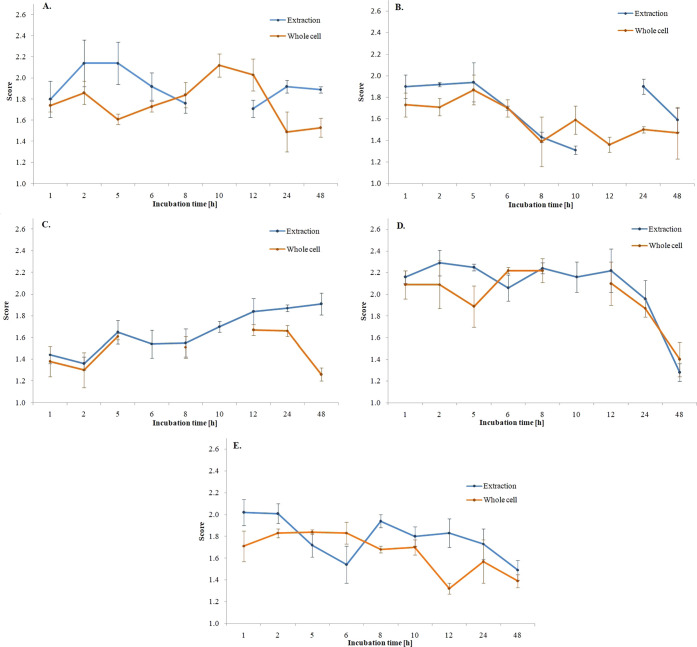
Graphs showing the dependence of average score values on the incubation time of bacteria (A—*B. licheniformis*, B—*P. simplex*, C—*Lysinibacillus sp*., D—*B. flexus*, E—*B. marisflavi*).

Based on the graph (Figure 1), it can be concluded that for sample 7NN, the best identification results were obtained after 2 h of incubation for the extract and 12 h for whole cells. For the 8NN, the best results were obtained after 5 h of incubation for both sample preparation methods. For the sample 11NN, it was complicated to determine the optimal incubation time due to the unclear species identification of microorganisms. For 12NN up to 1–12 h of incubation, scores ≥2.00 were obtained for bacterial extracts. Analyzing the results obtained for whole cells, the optimal incubation time is 8 h, although the species-level identification was obtained after only 1 h of incubation. For the 13NN samples, the best identification results were obtained after 1 h of incubation, both for extracts and whole cells.

**Figure 2:**
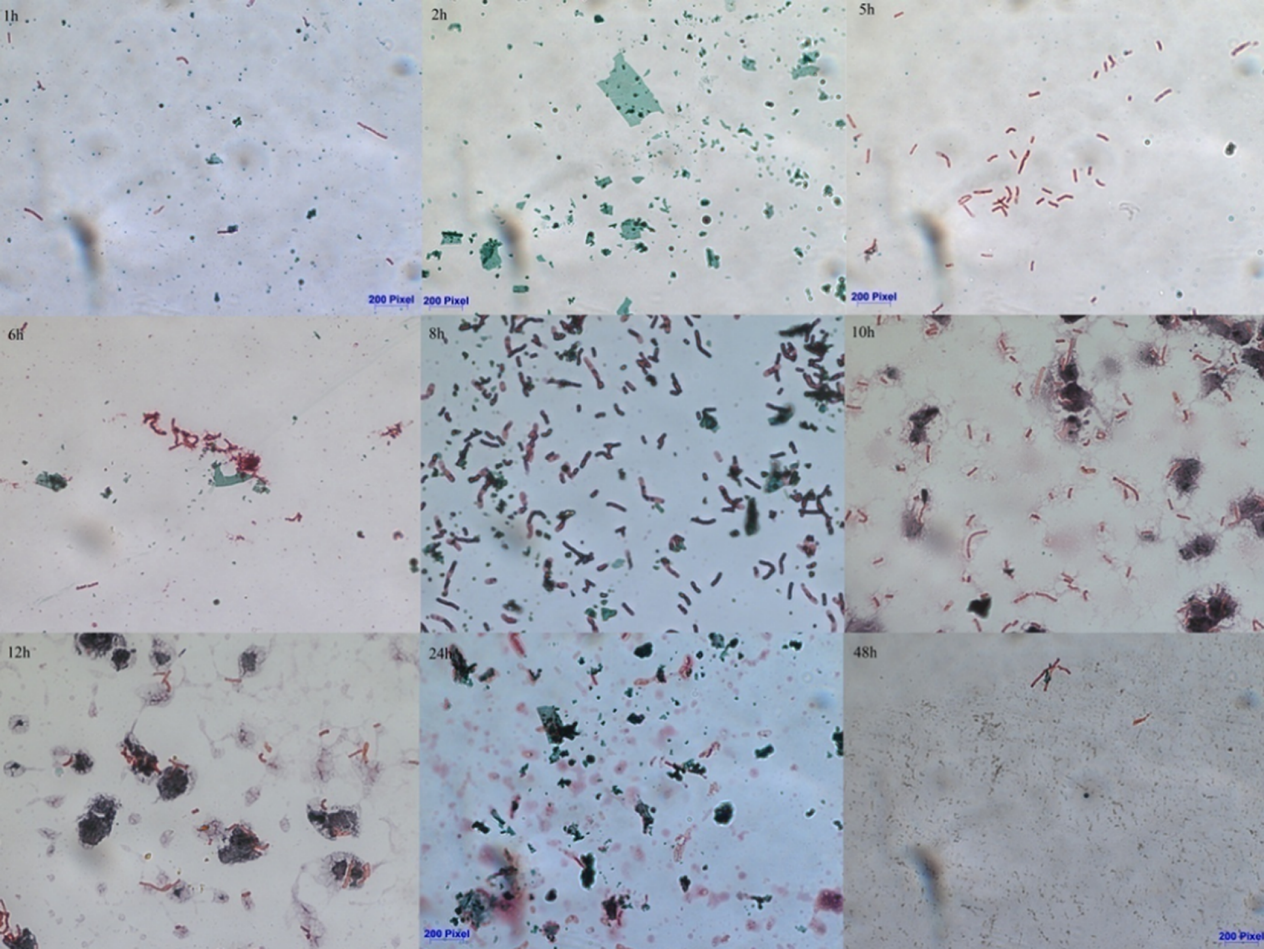
Microscopic photos of bacterial preparations after staining with the Schaeffer–Fulton method, showing endospores for the 8NN sample (green—spores, pink—vegetative cells).

Microscopic images taken after staining over time using the Schaeffer–Fulton method show that the incubation time of the bacteria influences the number of spores in the sample. Figure 2 shows changes in the number of spores for *P. simplex* (sample 8NN).

**Figure 3:**
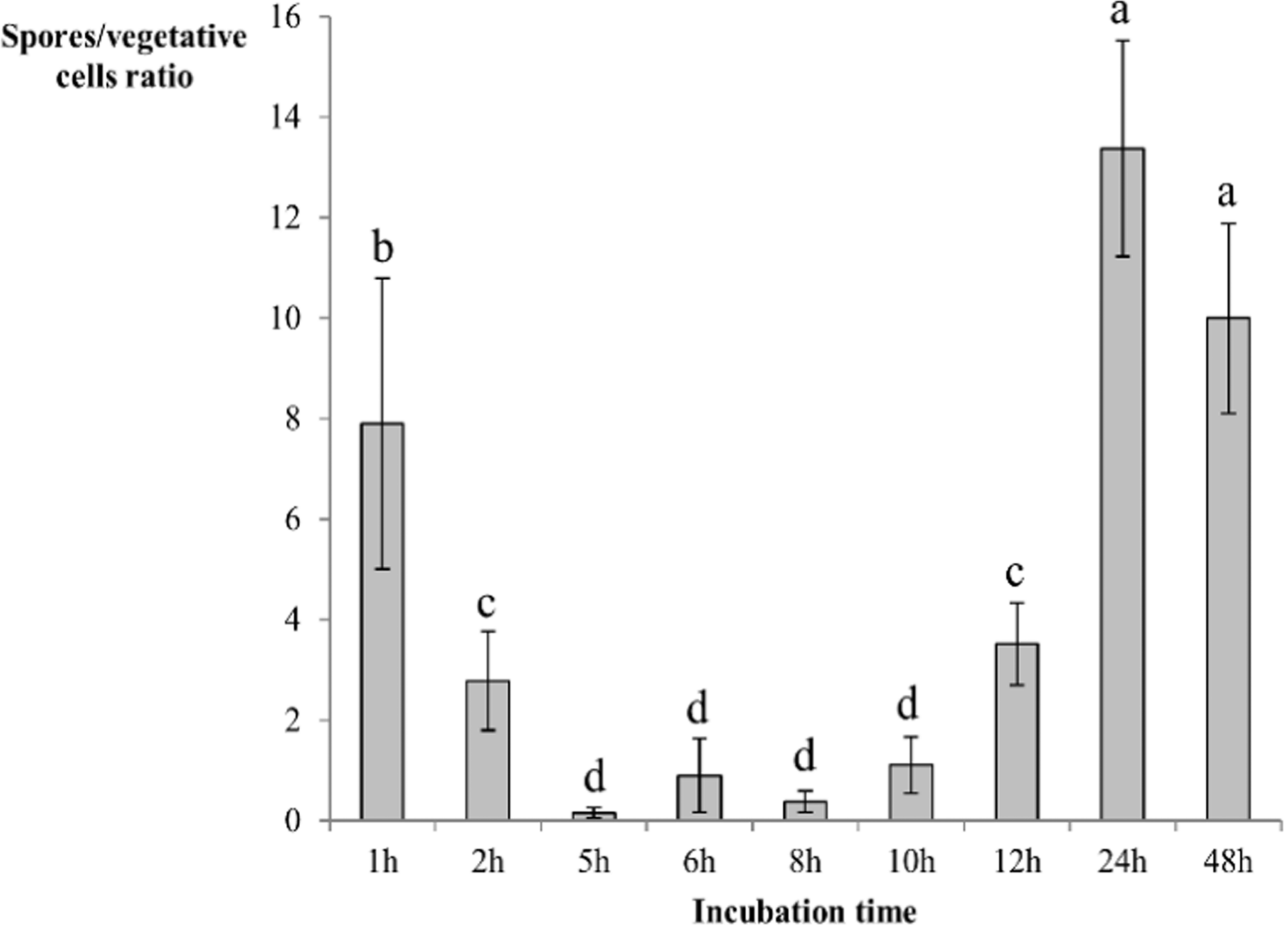
Spore production dynamics during incubation expressed as the ratio of spores to the number of vegetative cells calculated using optical microscopy as well as Gram and Schaeffer–Fulton staining techniques. Subsequent letters of the alphabet denote statistical differences between the results calculated using analysis of variance (ANOVA) and Fisher’s least significant differences (LSD) post hoc test in descending order.

On average, the ratio of spore to vegetative cell counts during successive incubation hours exhibited diverse values, specifically 4.5/1.8, 13.0/4.7, 1.7/10.5, 7.5/8.0, 3.3/8.5, 5.0/4.5, 13.5/3.8, 17.8/1.3, and 15.0/1.5, in that order. These observations suggest a notable fluctuation in the spore-vegetative cell relationship across the incubation period. Interestingly, the number of spores was observed to be more pronounced during the initial and concluding stages of the incubation period compared to the midpoint. This trend suggests an intriguing dynamic between spore formation and the progression of the incubation period, which merits further examination. Upon assessing the rates of spore production, expressed as the ratio of spores to vegetative cells (as depicted in Figure 3), we discovered that the minimum quantity of spores was recorded within the 5 to 8 h interval of the incubation period. This particular time frame is significant and can be considered the optimal incubation duration for our study. This is because we noticed a gradual upswing in the number of spores beginning from the 10 h mark of the incubation period. This gradual increase indicates the start of a prolific spore-forming phase, thus making the 5 to 8 h interval critical in terms of managing spore production for accurate identification of the bacteria. This understanding could potentially influence and improve the methodologies adopted for the identification of spore-forming bacteria.

### 2.4 MS Spectra Analysis

**Figure 4:**
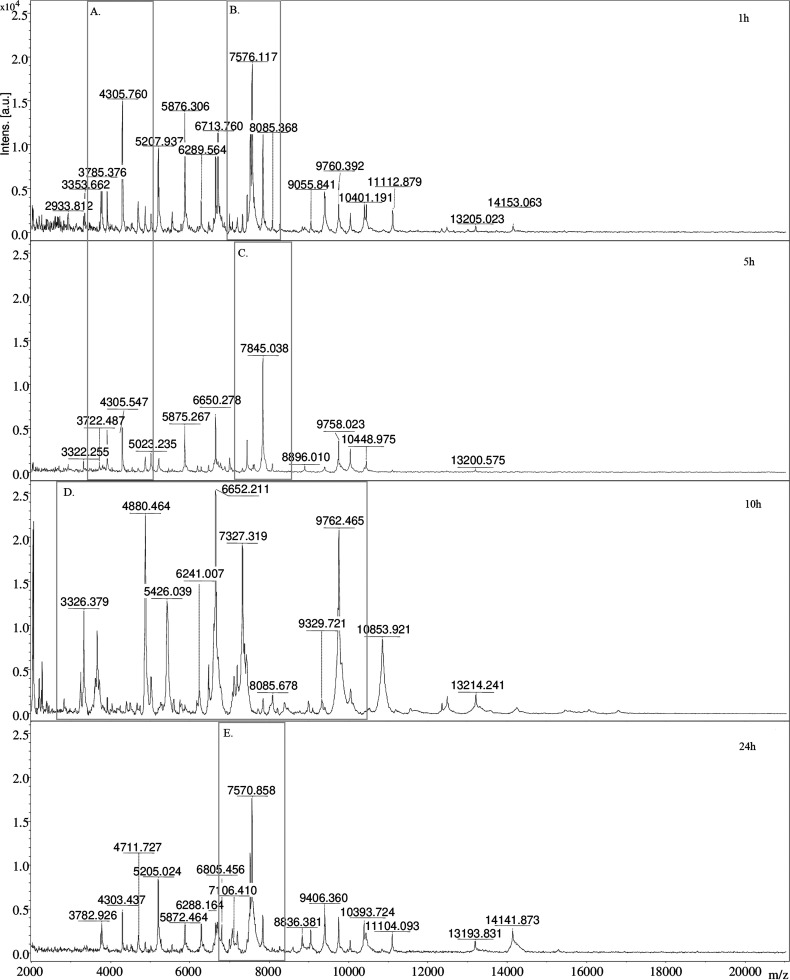
Comparison of MALDI-TOF MS mass spectra after 1, 5, 10, and 24 h incubation for 8NN sample.

To explain the differences in identification in relation to the incubation time, the mass spectra analysis was carried out for four selected times: 1, 5, 10, and 24 h. Figure 4 shows sample spectra obtained for the 8NN sample. Comparing the obtained spectra, it can be observed that in 1 h of incubation, the characteristic signals are 4305 (Figure 4A) and 7576 *m*/*z* (Figure 4B). After 5 h, it can be seen that the intensity of the 4305 *m*/*z* signal has decreased, while the 7845 *m*/*z* (Figure 4C) signal has appeared. According to the UniProt database, the 4305 *m*/*z* signal is due to the 50S ribosomal protein L36 or the sporulation protein YjcZ. The decrease in the intensity of the described signal after 5 h of incubation suggests that it is the protein responsible for sporulation. The signal 7576 *m*/*z* corresponds to the protein that builds bacterial spores. The signal that appeared after 5 h of incubation (7845 *m*/*z*) corresponded to the protein responsible for the germination of bacterial spores. This confirms the assumption that spore germination and an increase in the number of vegetative cells occur after this time. The signal 7576 *m*/*z* corresponds to the protein that builds bacterial spores.

After a 10 h incubation, the MS spectrum shows signals 3326, 4880, 6652, and 9762 *m*/*z* (Figure 4D), which, according to the UniProt database, are responsible for bacterial sporulation, which explains the decrease in the level of identification of microorganisms. A high signal intensity of 7570 *m*/*z* (Figure 4E), corresponding to the germination protein, is observed after 24 h of bacterial incubation.

**Figure 5:**
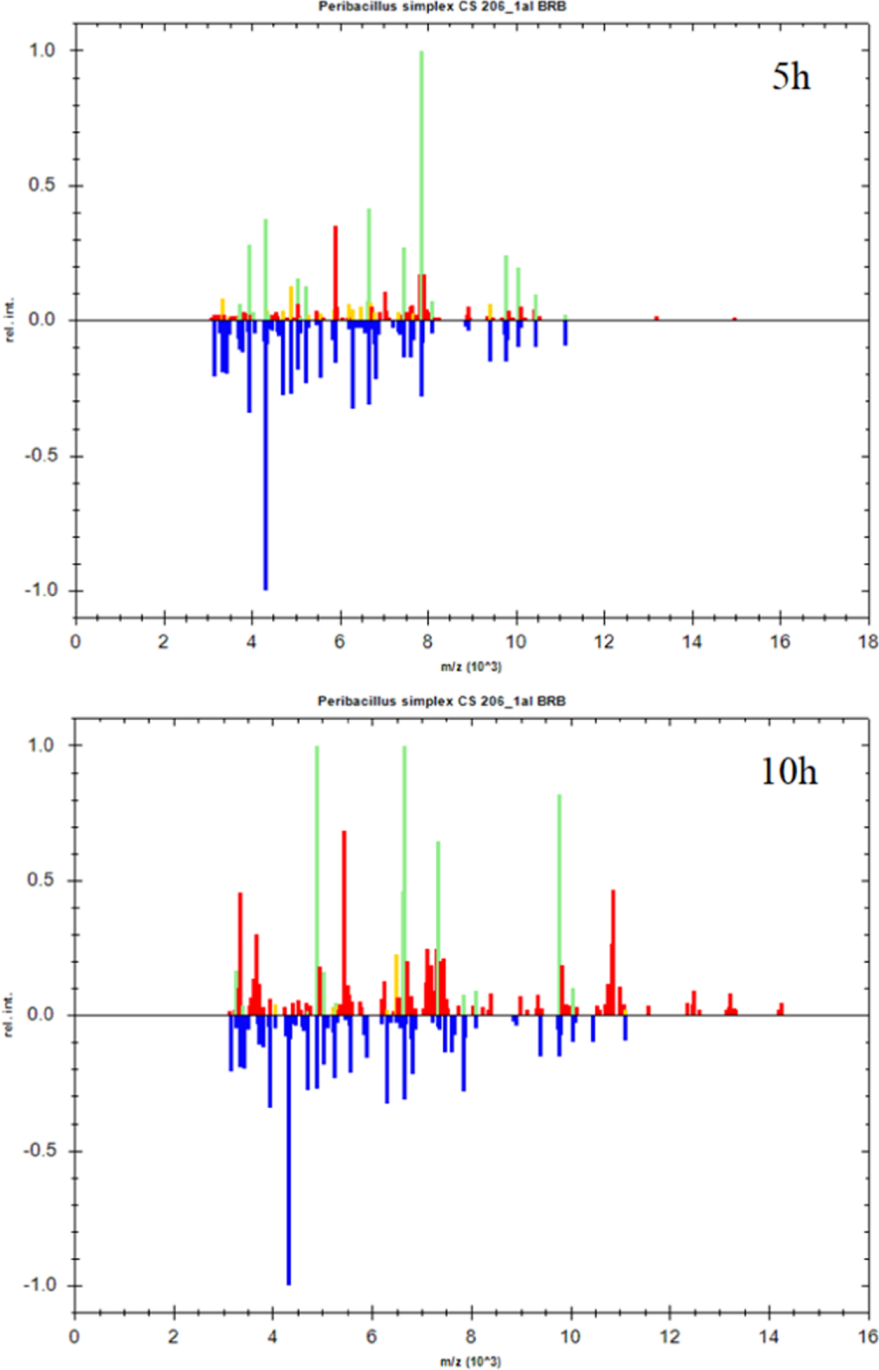
Comparison of the reference spectra with those obtained during the MALDI-TOF MS analysis of the 8NN sample after 5 and 10 h of incubation.

The reference spectra of *P. simplex* (8NN) were compared with the obtained spectra for 5 and 10 h (Figure 5). Green means species match (score ≥2), yellow—generic match (score 1.99–1.70), and red—no match (score <1.7). The spectrum obtained after 10 h of the incubation of bacteria shows the disappearance of signals from ribosomal proteins (on which the identification is based) in comparison with the spectrum obtained after 5 h of incubation. These results show a smaller number of ribosomal protein signals. The appearance of signals from proteins promoting sporulation negatively affects the identification of bacteria using the MALDI-TOF MS technique.

### 2.5 Statistical Analysis

**Figure 6:**
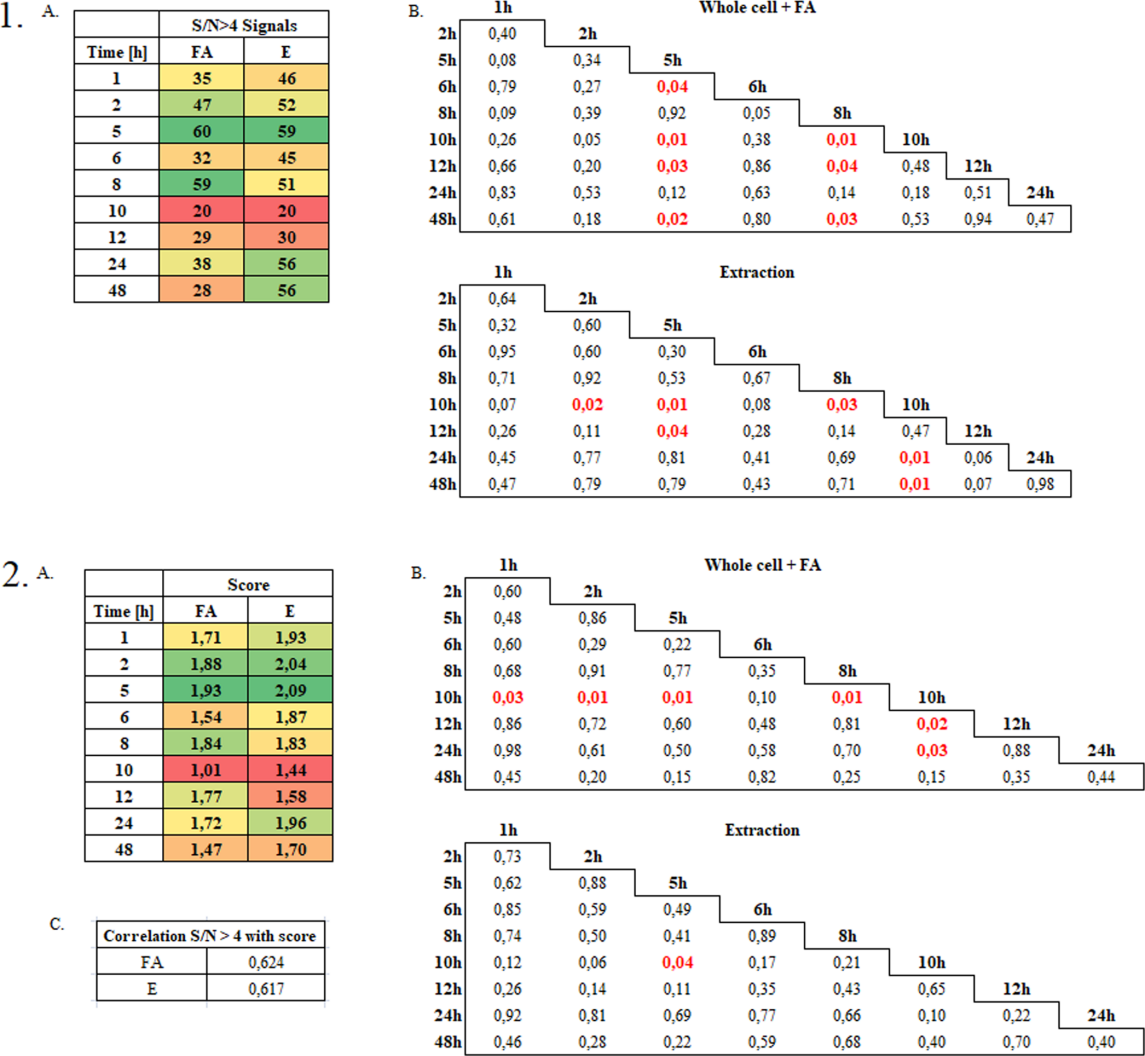
Significance tests of differences and existing correlations based on the number of signals (1) and the score value (2).

The results of statistical analyses performed with Statistica version no. 12 using ANOVA with Fisher’s least significant differences (LSD) post hoc test and Pearson’s linear correlation analysis are shown in Figure 6. When analyzing the data presented in Figure 6(1.) it was observed that the number of *S*/*N* ≥ 4 signals in the tested samples increased during the incubation up to 5 h and significantly decreased during 10 h of incubation, regardless of the mode—FA (whole cell + FA) or E (extraction)—3 times compared to 5 h of incubation. Although during the further incubation—12–48 h—a gradual increase in the number of acquired signals in the MS spectra was observed. Only in the case of the E mode, their value approach those recorded until 5 h of incubation.

Taking into account the statistical significance of differences in the number of signals, in most cases they concerned the 5 and 10 h incubation variants, where a significant increase and decrease in the number of signals was observed, respectively. Generally speaking, the most optimal incubation time in terms of the quality of the MS spectra and the identification of bacterial species was 5 h, while the time of 10 h turned out to be the critical point characterized by the poorest quality of the spectra and the level of identification.

The data presented in Figure 6(2.) allow us to conclude that the highest score value was recorded after 5 h of incubation, which then decreased to the lowest value after 10 h of incubation, although most of the differences were not statistically significant except for the score for the FA mode and 10 h of incubation. After the incubation longer than 10 h, the score value increases, but it reaches lower values than in the case of shorter incubation times—up to 8 h. For both modes, a high correlation (0.5 < *r* ≤ 0.7) of the *S*/*N* ≥ 4 number with the score value was noted.

## 3 Discussion

Spore-producing bacteria pose a significant risk to food safety and public health, as they are responsible for contamination and disease transmission in plants, animals, and humans. These bacteria form spores, highly resistant to chemicals, disinfectants, and various physical treatments, challenging their eradication. Consequently, rapid and accurate detection of spore-forming bacteria is a crucial objective in microbiology. The MALDI-TOF MS emerged as a vital technology in clinical microbiology laboratories due to its ability to rapidly identify microorganisms accurately. Despite its many advantages, the method faces challenges when detecting spore-forming bacteria. The presence of spores complicates the identification process, as the formation of a thick cell wall and the difficulties associated with protein extraction hinder the accurate analysis.

The thick cell wall of spores provides remarkable resilience, allowing them to survive extreme conditions such as high temperatures, radiation, and desiccation. This durability poses a problem for traditional bacterial identification methods, as the cell wall obstructs the release of intracellular proteins required for the analysis. Consequently, researchers must develop innovative strategies to overcome these challenges and improve detecting and identifying spore-forming bacteria using MALDI-TOF MS.

Potential avenues for enhancing the MALDI-TOF MS performance in spore-forming bacteria detection include optimizing sample preparation techniques, such as employing specialized extraction protocols or modifying incubation conditions. Researchers could also explore using alternative matrices and developing novel bioinformatics tools to interpret the mass spectra of spore-forming bacteria better.

Micro-biologists can significantly enhance food safety and quality control measures by addressing these challenges and refining the MALDI-TOF MS method for spore-forming bacteria detection. Ultimately, such advancements will contribute to the protection of public health by facilitating the rapid identification and mitigation of potentially harmful spore-forming bacterial contaminants in food and other environments.

Bacillus is ubiquitous and is one of the most widespread species with high phenotypic and genotypic diversity. *Bacillus* bacteria can be lethal pathogens used as biological weapons, cause opportunistic infections, contaminate food, and be used as probiotics.^21,22^ In addition, Bacillus species can produce a variety of extracellular enzymes such as amylase, protease, lipase, and lecithinase, so they can potentially grow and spoil a variety of food matrices.^23,24^ Reference spectra of many Bacillus species are available in commercially available databases such as Biotyper 3.0; however, due to the presence of endospores that mix with vegetative cells, the classification and identification of spore-forming bacterial strains using MALDI-TOF MS is complicated. It was demonstrated that the variable vegetative-endospore composition of bacterial cells has a direct impact on the identification of bacteria and that the proportion of SASP ranging from 8 to 20% is inconsistent, making accurate identification of bacteria of the *Bacillus* species even more difficult.^25−29^

Optimizing culture conditions is a straightforward and effective strategy for managing spore formation, which is vital for obtaining reliable results in bacterial identification studies. In our research, we employed two distinct preparation methodologies for MALDI-TOF MS analysis. The first method entailed a direct transfer process complemented by a formic acid coating, and the second approach utilized a process of extraction with ethanol and formic acid. In our comparison of these two techniques, we found that the overall impact on the identification outcomes of spore-forming bacteria was statistically insignificant. In other words, both methods yielded comparable results in terms of bacterial identification, which suggests that researchers can select either method based on other considerations such as resource availability, time efficiency, or convenience. Importantly, our findings align with existing literature on this subject. Previous research has indicated that the direct transfer method with a formic acid coating is highly effective for the identification of Gram-positive bacteria at a species level. The validation of this method in our own study further strengthens the argument for its use in bacterial identification processes, particularly those involving spore-forming bacteria. These insights contribute to a growing body of knowledge on culture optimization and sample preparation methods, informing future research and best practices in the field of microbial identification. The more we understand these processes and their impact on identification outcomes, the better we can refine our methodologies and improve the accuracy and efficiency of bacterial identification 09:35 PM. The growth rate directly affects the transformation of vegetative cells into endospores. For example, the research conducted by Shu and Yang proves the possibility of shortening the standard 24 h *Bacillus* incubation time to 12 h (solid medium).^30^ Our research shows that even a very short incubation (up to 1 h) in the liquid medium of the bacteria allowed the identification of *P. flexa* at the species level, and satisfactory identification results were obtained up to 24 h of incubation. The worst identification results were obtained for sample 11NN, which consisted of the bacteria of the genus *Lysinibacillus* (Table 3). Species identity could not be established as successive replicates gave a different species identification result. This may be due to the close relationship between *L. fusiformis*, *L. xylanilyticus*, and *L. pakistanensis*. Nevertheless, each of the analyzed samples showed the same relationship of decreasing score values with the length of the incubation time. Microscopic images (Figure 2) and statistical analysis (Figure 3) confirm the higher presence of spores in the first and last hours of incubation of the microorganisms. A similar phenomenon was reported by Chambers et al.^31^ and Shu et al.^30^ who demonstrated that endospores directly reduced the identification scores assessed by MALDI-TOF MS and disturbed the identification of *Bacillus* species. Statistical analyses (Figure 6) show that 10 h of incubation is the least favorable in terms of the identification quality while 5 h is the best. It is reported that the sporulation time for *B. subtilis* is 8–10 h, which confirms the weakest identification results after 10 h of incubation.^32,33^ Literature data indicate that spore germination and integumentary overgrowth in *Bacillus* bacteria typically occurred within 1–2 and 3–7 h, respectively.^34^ This is a supporting argument for why our results indicate that 5 h of incubation time gives the best identification results. Regarding the missing spectral data for *Lysinibacillus* at 6, 8, and 10 h of incubation, we would like to provide further clarification. While Figure 3 does indeed show that the population of bacteria is primarily formed by vegetative cells with a minimal number of spores during these time points, the sporulation process itself can pose challenges in obtaining clear spectral data. Obtaining spectra from spores is more challenging than from vegetative cells. However, it is worth noting that the sporulation process in bacteria involves a series of complex morphological and biochemical changes. During this transition phase, even if the majority of the cells are vegetative, the onset of sporulation can lead to alterations in the protein content and profile of the bacterial population. This can result in a heterogeneous mixture of proteins, which can complicate the extraction and ionization processes.

Furthermore, sporulating cells can produce a variety of proteins and other molecules that can interfere with the MALDI-TOF MS process. These molecules can either suppress the ionization of other proteins or produce additional peaks that can overshadow the signals from the main proteins of interest. As a result, even if the number of spores is low, the presence of these sporulation-related molecules can lead to a spectrum that is poor in signals, making it challenging to obtain clear and reliable data.

In our study, we observed that during the 6, 8, and 10 h incubation periods, the sporulation process, even though not dominant, had a significant impact on the quality of the spectra obtained. This led to the lack of data for these time points, as the spectra were not of sufficient quality for reliable identification using the Biotyper platform.

It is worth noting that the reproducibility and reliability of MALDI-TOF MS can be influenced by various factors, including protein extraction procedures and the system’s status in different centers, as highlighted by Rodríguez-Temporal et al. in their multicentre study on nontuberculous mycobacteria identification.^35^

Although the microorganisms described in the above study are mainly responsible for food spoilage, they may be related to the occurrence of infections in humans. For example, due to the co-infection of *B. subtilis* and *B. licheniformis*, La Jeon et al. found an atypical pattern of bacteraemia and mediastinitis in immune-compromised patients.^36^*B. licheniformis* can also cause foodborne illness, the symptoms of which are nausea, vomiting, diarrhea, and stomach cramps occurring 5–12 h after eating contaminated foods.^37^ Raviane discovered the presence of *Bacillus* on thermoplastic immobilization masks used during radiation therapy treatment. Moreover, the recovery of bacteria from stored masks after 4 weeks indicates the continued presence of dormant spores. This also means that they can be transferred to a patient wearing a contaminated mask during a treatment session.^38^ As the results obtained in the above study indicate, an approximately 5 h incubation of bacteria is the right time for spore germination and the development of vegetative cells. Spores are very resistant to any changes in the environment, which cannot be said about vegetative cells, which is why it is easier to fight them. The results obtained give hope for the possibility that the rapid identification of spore-producing microorganisms contamination control can help solve the problem of food contamination. Quick analysis, when vegetative cells prevail in the sample, can be used to opt for the drastic antibacterial therapy (pasteurization, detergents, UV, or appropriate antibiotics).

Nevertheless, additional steps must be taken to obtain a comprehensive dataset that enables the precise and rapid identification of spore-forming bacteria. In the future, efforts will be made to identify these microorganisms using lipidomics and metabolomics approaches combined with the MALDI-TOF MS technology. The aforementioned studies are considered pilot and preliminary investigations. Advancements in these areas could potentially facilitate the identification of clinically relevant spore-forming bacteria in the near future.

## 4 Conclusions

This comprehensive research significantly advanced our understanding of spore-forming bacteria identification using MALDI-TOF MS and the crucial factors that impact the accuracy and efficiency of this method. The study has thoroughly investigated the optimal incubation time, and sample preparation methods, and explored the potential integration of lipidomics and metabolomics approaches to improve bacterial identification.

The implications of the findings are vast and far-reaching, encompassing a range of applications such as food safety, clinical diagnostics, and environmental monitoring, where swift and precise identification of spore-forming bacteria is of paramount importance. By enhancing the capabilities of MALDI-TOF MS in clinical microbiology laboratories, the research contributes to the development of more effective strategies for detecting and managing bacterial contamination.

Moreover, this study emphasizes the need for ongoing research and innovation in the field of microbiology, particularly concerning spore-forming bacteria detection and identification. These advancements will ultimately lead to more robust tools and methodologies, ensuring public health and the safety of our food supply. Additionally, the research findings have the potential to influence policy-making, regulatory frameworks, and industry practices, driving a more proactive and science-based approach to managing the risks posed by spore-forming bacteria.

## 5 Experimental Section

### 5.1 Instrumentation

The identification of isolated microorganisms was performed using an MSP 96 target polished steel BC plate, microflex LT MALDI-TOF mass spectrometer (Bruker Daltonik, Bremen, Germany). For amplification by the polymerase chain reaction (PCR), a Master cycler pro-S thermocycler (Eppendorf, Hamburg, Germany) was used, gel electrophoresis was performed using a PowerPac power supply (Bio-RAD Laboratories, Hercules, CA), and a NanoDrop 2000c (Thermo Fisher Scientific, Wilmington, DE) was used to measure the concentration of the DNA. For the microscopic analysis, an Axio Observer D1 microscope (Carl Zeiss, Oberkochen, Germany) was used.

### 5.2 Chemical and Reagents

Tryptic Soy Agar (TSA) and Tryptic Soy Broth (TSB) (both from Sigma-Aldrich, Germany) were used for the bacteria cultivation. To obtain the bacterial protein extract, HPLC-grade water, formic acid (FA), acetonitrile (ACN), ethanol, and trifluoroacetic acid (TFA) (all from Sigma-Aldrich, Germany) were applied. For the MALDI-TOF MS analysis, α-cyano-4-hydroxycinnamic acid (HCCA) (Sigma-Aldrich, Switzerland) and Bacterial Test Standard (BTS) (Bruker, Germany) were used. For the DNA isolation, the QIAamp DNA Microbiome Kit reagents (Qiagen, Germany) were used, while RNase-Free Water (Qiagen), agarose (Merc, Germany), 50×TAE buffer, and ethidium bromide (both from AppliChem, Germany) were used for electrophoresis. Microbiological preparations were stained with crystal violet, Lugol’s reagent, safranin (all from aqua-med ZPAM-KOLASA, Poland), and malachite green (Sigma-Aldrich, Germany).

### 5.3 Bacteria Isolation from a Food Sample

The bacteria were isolated from cow’s milk, sauerkraut, and pickled cucumber samples using the serial dilution method, culturing on TSA plates. Bacteria were incubated in aerobic conditions at 37 °C for 24 h.

### 5.4 Sample Preparation of Bacteria

**Figure 7:**
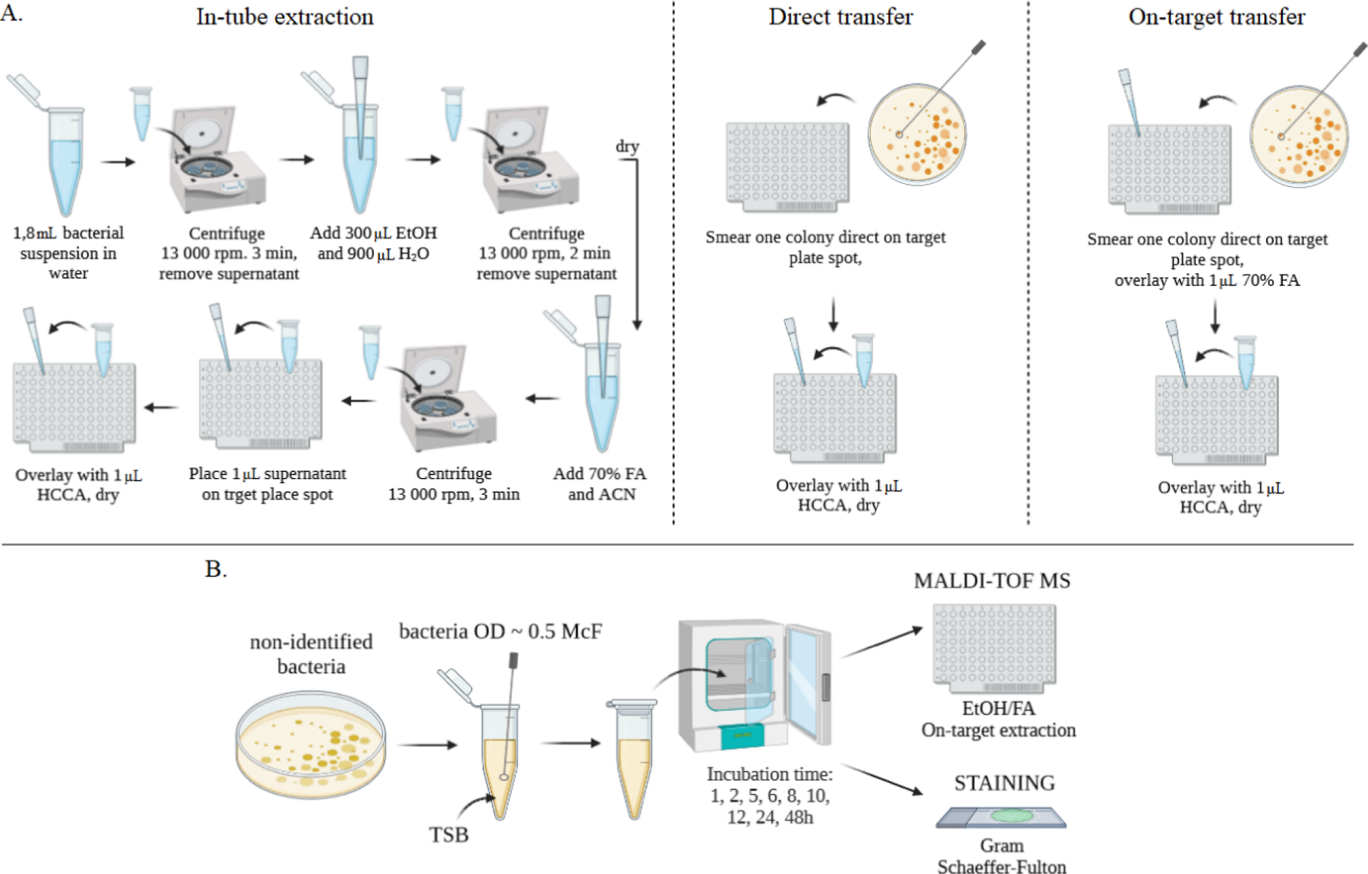
Procedure of sample preparation (A) and selection of incubation parameters for the spore-forming bacteria (B).

Three sample preparation methods were used: in-tube extraction, on-target extraction, and direct colony transfer^28^ (Figure 7A). In the first method, 1.8 mL of the bacterial suspension was centrifuged at 13,000 rpm for 5 min, and then 300 μL of distilled water and 900 μL of ethanol were added to the pellet. The samples were centrifuged at 13,000 rpm for 2 min, and the pellet was dried. An appropriate amount of formic acid and acetonitrile was then added, and the resulting suspension was centrifuged at 13,000 rpm for 2 min. 1 μL of the resulting supernatant was applied to an MSP 96 Polished Steel BC target plate, dried at room temperature, and covered with 1 μL of α-cyano-4-hydroxycinnamic acid (HCCA) matrix solution (10 mg HCCA/1 mL solvent solution: 50% ACN, 47.5 H_2_O and 2.5% TFA). In the on-target extraction method, a single bacterial colony was smeared directly onto the target plate, overlaid with 1 μL of 70% formic acid, and allowed to dry at room temperature. Then, 1 μL of HCCA matrix solution was applied. The last method, direct colony transfer, is a method analogous to on-target extraction, except that the smeared bacterial colonies were covered directly with the HCCA matrix solution.

### 5.5 Bacteria Identification Based on the MALDI-TOF MS Approach

The spectral data collection process involved the use of a microflex LT MALDI-TOF mass spectrometer, while processing of the collected spectra was facilitated via the MALDI-Biotyper 3.0 platform (Bruker Daltonics, Inc.). Each of the biological samples (*n* = 3, derived from a single culture) was subjected to triplicate analysis to ascertain consistency and accuracy of the findings. Furthermore, for each technical replicate, a series of three separate spectra were manually collected (*m* = 3), with each spectrum being composed of three times 1500 single-shot spectra collected in triplicate. The analysis encompassed the use of linear mode and positive ion mode, with ion source 1 set at 20 kV, focusing voltage at 7 kV, and detector voltage at 2.65 kV. The delayed extraction time was fixed at 230 ns, with a detection quality range between 2000 and 20,000 Da. Spectra calibration was accomplished via a quadratic calibration algorithm, as provided by BTS. The recorded spectra were managed through FlexControl (Bruker Daltonics, Inc.), and the ensuing data were analyzed via FlexAnalysis (Bruker Daltonics, Inc.). Prior to the analysis, each spectrum was subjected to a smoothing procedure through the Savitsky-Golay method (width 2 *m*/*z*, cycles 10), followed by baseline corrections via the TopHat algorithm (signal-to-noise threshold 2; peak detection algorithm–centroid), as recommended by the software supplier (Bruker Daltonik GmbH, Bremen, Germany). BTS (Bruker Bacterial Test Standard, Bruker Daltonik GmbH, Bremen, Germany) was used for spectra calibration, adhering strictly to the manufacturer’s instructions. Post the abbreviated incubation times of 7 and 9 h, we used both standard and mixed-detection modes for the bacterial identification phase. The MALDI Biotyper 3.0 system calculates the likelihood of accurate identification by comparing the entire protein profile obtained with the reference database. This is illustrated in a point index and graphical index, with respective values indicating the reliability of microorganism identification, either at the species or genus level.

### 5.6 Bacteria Identification Based on Sequencing of the 16S rDNA Gene

The genomic DNA according to the protocol was provided by the manufacturer. Then, a PCR reaction was performed to amplify the 16S rDNA region using universal primers complementary to the bacterial 16S rDNA (27F: 5-AGAGTTTGATCMTGGCTCAG-3 and 1492R: 5-GGTTACCTTGTTACGACTT-3). The amplification was carried out under the conditions described by Pomastowski et al.^7^ A Mastercycler pro-S thermocycler was used for all PCR reactions. The concentration and purity of the obtained amplicons were quantified by spectrophotometry. Additionally, the quality of the obtained DNA was analyzed using agarose gel electrophoresis, staining the genetic material with ethidium bromide. The PCR products were sent to Genomed (Warsaw, Poland) and were sequenced via the Sanger dideoxy method using the same primers. Finally, contigs were assembled via BioEdit Sequence Alignment Editor ver. 7.2.5^39^ and then consensus sequences were compared to known 16S rDNA reference sequences in the National Center for Biotechnology Information database RNA reference sequences (RefSeq RNA) using the Basic Local Alignment Search Algorithm tool.

### 5.7 Selection of Incubation Parameters for the Spore-Forming Bacteria

Selected bacteria that could not be identified after 24 h incubation in the TSA medium were incubated for 1, 2, 5, 6, 8, 10, 12, 24, and 48 h in the TSB liquid medium. Each sample was analyzed for extracts and whole bacterial cells treated with formic acid (Figure 7B). One sample was analyzed in triplicate, the average was taken, and the standard deviation was calculated. Additionally, Gram staining and Schaeffer–Fulton staining to detect the presence of spores were performed. Pictures of microscopic preparations were taken using an Axio Observer D1 fluorescence microscope with a camera and Axiovs40 V 4.8.2.0 software.

### 5.8 Statistical Analysis

Tests of significance of differences and existing correlations were carried out using Statistica version no.12 (StatSoft, Inc.) using ANOVA with Fisher’s last significant differences (LSD) post hoc test and Pearson’s linear correlation analysis.
